# Habitat Type-Based Assemblage and Distribution Prediction of Small Mammals and Chigger Mites (Acari: Trombiculidae) in Chuncheon City, Republic of Korea

**DOI:** 10.3390/ani14233433

**Published:** 2024-11-27

**Authors:** Kiyoon Kim, Jusun Hwang, Kyungmin Kim, Kwangbae Yoon, Daehyun Oh, Yungchul Park

**Affiliations:** 1 College of Veterinary Medicine, Chungbuk National University, Cheongju 28644, Republic of Korea; anikest@naver.com; 2 Division of Forest Science, Kangwon National University, Chuncheon 24341, Republic of Korea; jusunhwangl@aware.kr; 3 Interdisciplinary Program of Eco Creative, Ewha Womans University, Seoul 03760, Republic of Korea; kyungminkim0829@gmail.com; 4 National Institute of Ecology, Yeongyang 36531, Republic of Korea; 5 Department of ICT convergence, Hansei University, Gunpo 15852, Republic of Korea

**Keywords:** small mammals, *Apodemus agrarius*, chigger mite, habitat, MaxEnt

## Abstract

While urbanization leads to habitat loss for medium and large mammals, small mammals can survive in fragmented habitats. As they are known to be the primary hosts of chigger mites (Acari: Trombiculidae) that transmit scrub typhus, their habitat can be considered the primary distribution area for chigger mites. This study aims to examine the distribution of small mammals and chigger mites in four habitat types and analyzed species richness, mean intensity (MI), dominance, and infestation rate (IR). A total of six small mammal species were captured, 76.8% of which were identified as *Apodemus agrarius*. Species richness of small mammals was highest in forests, followed by ecotones, grasslands, and agricultural lands. *Apodemus agrarius* accounted for 61% of the captures in forests, 80% in ecotones, 84% in grasslands, and 50% in agricultural lands, highlighting its varying dominance across habitat types. The MI of chigger mites was higher in grasslands (215.41 ± 20.70) and ecotones (171.67 ± 30.33) and lower in forests (76.67 ± 32.11). The MI of chigger mites was higher in the fall than that in the spring, and according to the MaxEnt model, they had a narrow, dense distribution in the fall and a wide distribution in the spring. These results suggest that inducing increased species richness of small mammalian hosts by habitat management and vegetation diversification may contribute to a reduction in the MI of chigger mites.

## Simple Summary

This study examines the relationship between small mammals and the chigger mites (Acari: Trombiculidae) that parasitize them in different habitat types within a region of South Korea. Small mammals are important hosts for chigger mites, which are vectors for diseases such as scrub typhus. We investigated how the distribution of small mammals and their parasitic mites varies across forests, grasslands, agricultural lands, and ecotones. Our findings suggest that grasslands and ecotones near human settlements have the highest abundance of small mammals, particularly *Apodemus agrarius*, and consequently, higher rates of mite infestation. In particular, the density of *A. agrarius* increased significantly in the fall, leading to a sharp rise in mite infestation rates and mean intensity. By understanding how habitat characteristics influence the abundance of small mammals and their parasites, this research highlights the importance of habitat management strategies to reduce the spread of mite-borne diseases to humans.

## 1. Introduction

Urbanization has reduced and fragmented wildlife habitats, resulting in bio-homogenization and significantly impacting wildlife populations [1,2,3,4]. Habitat fragmentation creates small, disconnected habitats, resulting in habitat loss for medium- to large-sized mammals and interior species with wide home ranges [5,6]. In contrast, small mammals with relatively narrow home ranges can inhabit urban areas such as parks, as well as ecotone created by habitat fragmentation [7,8,9]. They are often found living in close proximity to human settlements. This indicates the need for an approach that considers the role of small mammals as hosts and their areas of activity in the dispersal pathways of arthropod-borne disease vectors reaching humans.

Chigger mites belong to the family Trombiculidae, and of the 60 species known to exist in the Republic of Korea (ROK) [10], eight have been identified as vectors of scrub typhus [11]. Chigger mites feed exclusively on host bodily fluids [12], and their emergence peaks in spring and fall [13]. The preferred hosts are small mammals which are widely distributed throughout the ROK [12,14]. The abundance of chigger mites is affected by the density of their hosts [15], as the likelihood of feeding on host bodily fluids is higher when host density is higher due to increased opportunities for contact [16]. Small mammals are known to maintain higher densities within their habitats than medium and large mammals [17,18], leading to higher opportunities for contact with ectoparasites. The density of small mammals affects the accumulation and transmission of ectoparasites within their habitats, which in turn affects ectoparasite populations and abundance [19,20]. Therefore, among the various vegetation types that can accommodate chigger mites, such as forests, grasslands, and agricultural lands [21], those with high populations of their primary host, small mammals, are likely to have high abundances of chigger mites.

Since the ectoparasite as a vector has limited mobility, its dispersal range is considered to be the distance traveled and area of activity of the host [22,23,24,25]. Unlike medium- and large-sized mammals, which may also inhabit areas near human settlement depending on the context, small mammals are more widely dispersed throughout urban lands, agricultural lands, and even human settlements [17,26,27]. Additionally, pathogen carriage rates of small mammal ectoparasites are not known to differ between urban and non-urban lands [28]. Small mammals which live near human settlements act as hosts and transmit a various of pathogen to humans [20,29]. Small mammals should be considered key hosts that can influence the dispersal of chigger mites, especially those that use small mammals as primary hosts. Therefore, understanding the habitat preferences and population dynamics of small mammals is essential for predicting the distribution and abundance of ectoparasites such as chigger mites.

Complex interactions among hosts, ectoparasites, and environmental factors contribute to human exposure to disease vectors. Thus, a comprehensive understanding of these interactions is crucial not only for preventing human–vector contact but also for advancing epidemiological insights [30,31]. Despite the importance of these ecological dynamics, in the ROK, most studies on small mammals have primarily focused on rodents and their habitats within specific areas, such as fire-affected sites, afforestation areas, forest roads, and national parks [32,33,34,35,36]. Research on habitat preferences of small mammals remains limited. Additionally, studies on vectors parasitizing small mammals have largely been restricted to pathogen detection in chigger mites (Trombiculidae), ixodid ticks (Ixodidae), and fleas (Ctenophthalmidae) [37,38,39,40,41,42], as well as distribution studies focused on host species [43,44,45,46,47].

The hypotheses of the study were as follows. The assemblage of small mammals inhabiting different habitat types, such as agricultural lands, grasslands, the ecotones between forests and grasslands, and forests, will vary. Since ectoparasite abundance is influenced by host assemblage [17,47], the abundance of chigger mites will be higher where high populations of small mammals exist. This study aimed to determine the community and abundance of chigger mites associated with small mammal and host species in different habitat types and predict variation in the seasonal distribution of the hosts and parasitic chigger mites.

## 2. Materials and Methods

### 2.1. Ethics

This study received approval from the Institutional Animal Care and Use Committee (IACUC) at Korea University (KUIACUC#2016-49) and was conducted in compliance with the regulations of the Wildlife Protection and Management Act. Trapping permits were obtained from the district government (Chuncheon city). Specific protocols for live-trapping were followed in accordance with the guidelines of the American Society of Mammalogists for the use of wild animals in research and education [48]. Captured animals were euthanized by a veterinarian following procedures recommended by the American Veterinary Medical Association (AVMA) guidelines [49].

### 2.2. Study Area

**Figure 1:**
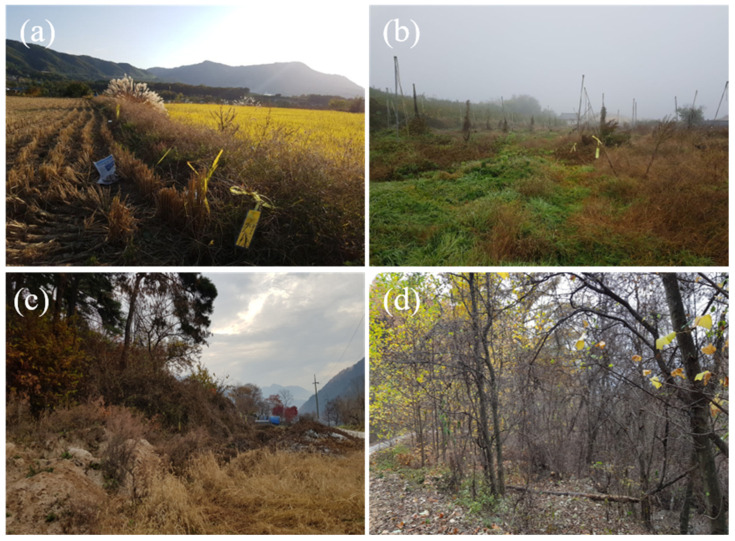
Habitat type in the study sites ((**a**): agricultural land, (**b**): grassland, (**c**): ecotone, (**d**): forest).

**Figure 2:**
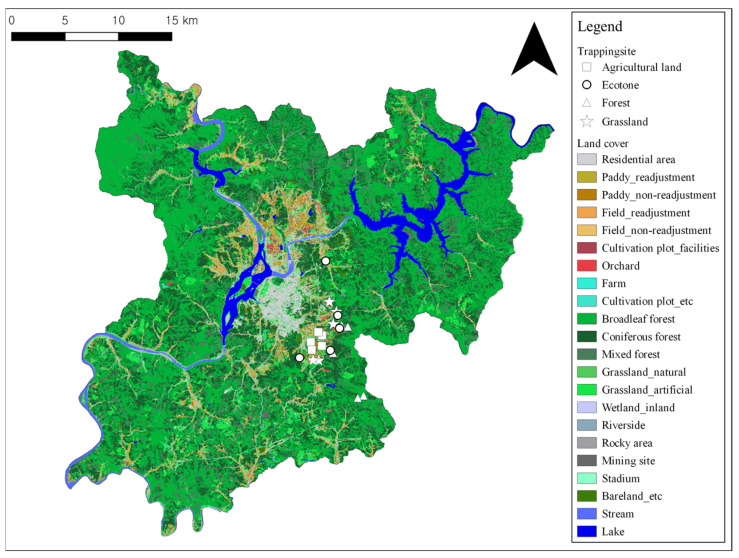
Small mammal capture sites in Chuncheon city (5 sites for each habitat type).

The study was conducted in Chuncheon city, Gangwon-do. According to the land cover map, the total area of Chuncheon city (1116 km²) comprises 75.79% forest, 7.09% grassland, 5.54% agricultural land, 5.50% water bodies, 3.15% urban areas, 2.13% bare patches, and 0.80% wetlands. Habitat types were classified as follows: agricultural lands, which included areas where crops such as peppers and sesame had been harvested in fall or were being cultivated in spring; grasslands, which included fallow fields with annual herbaceous plants, such as ragweed, or open grassy areas near residential areas; ecotones, which were defined as areas where herbaceous and shrub vegetation mix at the transition between mountains and grasslands; and forests, which were classified as mixed forest areas with over 60% tree canopy cover, consisting of both coniferous and broadleaf trees (Figure 1). Candidate sites for each vegetation type were identified and field-verified for consistency with the land cover classification. Small mammals were captured at 5 sites per vegetation type, for a total of 20 sites (Figure 2).

### 2.3. Capturing Small Mammals

Small mammal capture sessions were conducted during the fall (September to November 2017) and spring (March to May 2018) seasons. Biscuits (ACE, Haitai confectionery & foods, Seoul, Republic of Korea) were used as bait in Sherman traps (LFAD folding live capture trap, H.B.Serman traps Inc, Florida, USA), and cotton was placed inside the traps to reduce in-trap mortality from low temperatures. This measure was intended to keep the host alive until euthanasia to prevent ectoparasites from detaching due to host death. Twenty traps per site were set at 5 m intervals in a straight line of plots, following the line capture method recommended for small mammal community composition surveys [48], and the locations of all traps were recorded as coordinates. The captures were conducted on a four-day schedule, three times per area, with traps set from 4 to 5 pm before sunset and collected at 9 am after sunrise the following morning. Each of the four vegetation types was visited 15 times, with 20 Sherman traps set per visit, resulting in 1200 trap nights per season. This procedure was conducted in both spring and fall, yielding a combined total of 2400 trap nights. Captured small mammals were identified to species based on external morphology [50], and additional information was recorded for each individual, including body length, weight, and sex, which were measured to aid in identification.

### 2.4. Collecting and Identifying Ectoparasites

To collect and identify chigger mites parasitizing small mammals, captured small mammals were placed in a plastic container of water in a windless hood and suspended with their heads about 5 cm above the top of the Petri dish for 24 h to allow ectoparasites that had escaped from the carcass to fall into the Petri dish of water. Ectoparasites in the Petri dish were categorized into families based on external morphology and identified according to morphological characteristics; however, Anoplura could not be identified beyond the suborder level due to limitations in morphological features. [38,41,51,52]. Collected chigger mites were sorted by host, with half of the total individuals designated for species identification. For specimen preparation, 10 to 15 chigger mites were placed on a slide, mounted with a drop of PVA solution (56% polyvinyl alcohol, 22% lactic acid, 22% phenol solution), and positioned using a sterile needle with the dorsal and ventral surfaces facing directly upward, after which a cover glass was applied. The prepared chigger mite specimens were identified to the species level based on morphological characteristics [52] under a biological microscope (BX43, Olympus, Tokyo, Japan) at 400x magnification.

### 2.5. Data Analysis

Small mammal and ectoparasite species data cluster analysis was performed using SPSS (IBM, Version 26). The normality test was performed using the Shapiro–Wilk test. As the test revealed non-normality in the data collected, a Kruskal–Wallis analysis was performed to determine the significance of differences in small mammal capture and ectoparasite infestation rates among habitats. Thereafter, the Mann–Whitney with Bonferroni correction method was used to analyze whether significant differences were present among the four habitats. The significance level for the Kruskal–Wallis analysis was set at *p* < 0.05, and the significance level for the Mann–Whitney post hoc test was set at *p* < 0.0083 (0.05/6). The significance of seasonal differences was analyzed with the Mann–Whitney U test (*p* < 0.05). A Spearman’s correlation test was performed to analyze the correlation between the abundance of the dominant small mammal species in captivity, the dormouse, and the abundance of the chigger mite species. The average chigger mite infestation level of infected small mammals, analyzed by individual and by vegetation type, was presented as mean intensity (MI) in the format of average ± standard error [53]. Infestation rate (IR) refers to the proportion of captured individuals infested with ectoparasites. Capture rate indicates the number of small mammals captured per trap night, and dominance rate represents the proportion of a particular species within the community of captured small mammals. The community of small mammals was represented using the Shannon–Weaver diversity index (H’) [54], species richness index (RI) [55], and evenness index (E’) [56]. The Euclidean distance (in meters) from each small mammal trapping site, categorized by vegetation type, to the residential area (classified as a land cover type) was calculated as the shortest distance from each trap to the residential area using ArcMap 10.8.1 and was presented as the mean ± standard error for each vegetation type.

### 2.6. Modeling

To predict the distribution of small mammals and their parasitic chigger mites, MaxEnt (American Museum of Natural History, Version 3.4.4) was used. Using ArcMap 10.8.1, the classified land cover map of the study area, Chuncheon city (https://egis.me.go.kr/, accessed on 22 September 2022), was reclassified into 22 land cover types at a resolution of 10 m. The coordinates of the small mammal traps and the shortest Euclidean distance to each land cover type were measured and used as variables to predict the characteristics of the capture sites of individuals (Table 1). Given that the behavioral patterns of small mammals can vary by season, the coordinates of small mammals were categorized by season and used as input variables. The weight was applied by duplicate entry of the coordinates of the host capture site and was weighted by the number of chigger mites collected per host. The model was trained with five iterations of the cross-validation run type, and the average of these was used as the final model. Variables with percent contribution and permutation importance values of 5 or higher were selected for the model. The goodness of fit of the final model was assessed by area under the receiver operating curve (AUC) and accurate scale statistic (TSS) using R software (version 4.2.1), and a model with AUC and TSS of 0.6 or higher and 0.4 or higher, respectively, was considered adequate. [57].

## 3. Results

### 3.1. Small Mammal Communities by Habitat and Season

Six species of small mammals were captured during the 2400 trap nights: Striped field mouse (*Apodemus agrarius* Pallas, 1771), Ussuri white-toothed shrew (*Crocidura lasiura* Dobson, 1890), Asian lesser white-toothed shrew (*Crocidura shantungensis* Miller, 1901), Korean red-backed vole (*Craseomys regulus* Thomas, 1907) Eurasian harvest mouse (*Micromys minutus* Pallas, 1771), and Siberian chipmunk (*Eutamias sibiricus* Laxmann, 1769). The total number of captured individuals was 185, with 142 (76.26%) Striped field mouse (SFM), 19 (10.27%) Ussuri white-toothed shrew (UWS), 10 (5.41%) Asian lesser white-toothed shrew (ALWS), 6 (3.24%) Korean red-backed vole (KRV), 4 (2.16%) Eurasian harvest mouse (EHM), and 4 (2.16%) Siberian chipmunk (SC).

By habitat, 84, 55, 34, and 12 individuals of small mammals were captured in grasslands, ecotones, forests, and agricultural lands. More small mammals were captured in the fall than the spring, with 133 in the fall and 52 in the spring.

Of the 142 individuals of SFM captured, 71 (50%) were captured in grasslands, 44 (30.9%) in ecotones, 21 (14.7%) in forests, and 6 (4.2%), the least, in agricultural areas. SFM, UWS, and ALWS were captured in all habitats, EHM was captured in all but agricultural areas, and KRV and SC were captured only in ecotones and forests (Table 2).

Species richness was highest in forests, followed by ecotones and grasslands (excluding agricultural lands). Despite having the largest number of small mammal individuals captured, grasslands had the lowest diversity index, with only four species, and SFM accounted for 71 individuals (84%). Contrastingly, forests and ecotones with six species had relatively high H’ indices. The Striped field mouse, the small mammal species captured across all four habitats and in both seasons, was used to analyze seasonal and habitat-based variations in capture and infestation rates (Table 3). The capture rate was higher in the fall than in the spring, but no significant difference in infestation rate (*p* < 0.05) was observed. The capture rate by habitat was highest in grasslands (11.82%) and lowest in agricultural lands (1.00%), as was the case overall, and all showed significant differences (*p* < 0.0083), whereas the ectoparasite infestation rate did not show significant differences. The within-community dominance of SFM was significantly higher in grasslands (84.52%) and ecotones (80.00%) and lower in forests (61.76%) and agricultural lands (50.00%) (*p* < 0.0083).

**Figure 3:**
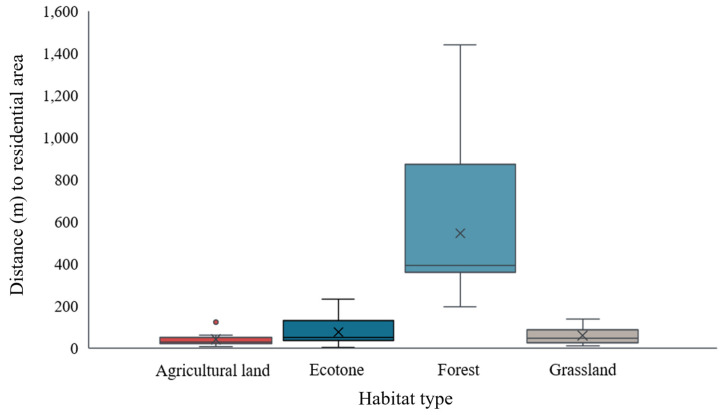
Euclidean distance (m) to the residential area of captured small mammals in each habitat type.

The shortest distances from small mammal capture sites to urban land areas were found in agricultural lands (41.4 ± 29.9), grasslands (59.9 ± 41.1), ecotones (76.5 ± 63.3), and forests (546.7 ± 351.1), with capture sites in forests being significantly more distant (Figure 3) (*p* < 0.05).

### 3.2. Community Structure of Chigger Mites Parasitizing Small Mammals by Habitat and Season

**Figure 4:**
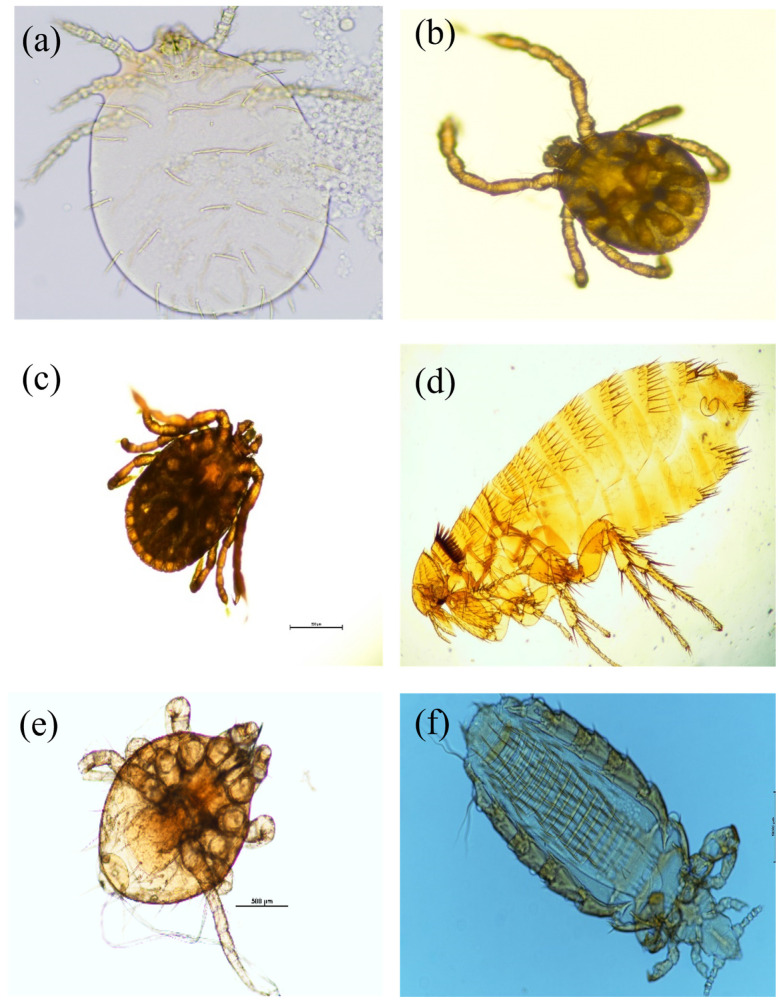
The ectoparasites (four families and one order) parasitic on small mammals ((**a**): *Leptotrombidium orientale* of Trombiculidae, (**b**): *Haemaphysalis* sp. of Ixodidae (larva), (**c**): *Haemaphysalis* sp. of Ixodidae (nymph), (**d**): *Neopyslla speciallis* of Ctenothalmidae, (**e**): Laelapidae, (**f**): Anoplura).

Four families and one order of ectoparasites were cataloged from 152 infested small mammals (Figure 4). The family Trombiculidae parasitized the most individuals with 126 (84.2%), followed by the family Laelapidae with 74 (48.7%), the family Ixodidae with 62 (40.8%), the family Ctenophtalmidae with 40 (26.3%), and the suborder Anoplura with 14 (9.2%). All four ectoparasite families and one order were detected on SFM, and four families were detected in KRV. Among ectoparasites, Trombiculidae and Ctenophtalmidae parasitized all six small mammal species as hosts. Ixodidae parasitized five species, and Laelapidae and the suborder Anoplura parasitized two species of the small mammal species, respectively. The infestation rate (IR) and mean intensity (MI) of parasites were the highest in the family Trombiculidae and the lowest in the suborder Anoplura (Table 4).

Based on morphological characters, species identification of Trombiculidae revealed a total of nine species: *Leptotrombidium orientale* Schluger, 1948, *Leptotrombidium palpale* Nagayo et al., 1919, *Leptotrombidium pallidum* Nagayo et al., 1919, *Leptotrombidium zetum* Traub, 1958, *Neotrombicula gadellai* Kardos, 1961, *Neotrombicula japonica* Tanaka et al., 1930, *Neotrombicula nagayoi* Sasa et al., 1950, *Neotrombicula tamiyai* Philip et al., 1950, and *Euschoengastia koreanensis* Jameson and Toshioka, 1954. All identified chigger mite species were collected from SFM, whereas five species were collected from UWS, two species from ALWS, four species from EHM, five species from KRV, and three species from SC. Furthermore, five hosts, with the exception of SFM, were either not captured in both spring and fall or chigger mites were not collected in both seasons. Therefore, a cluster analysis was performed on chigger mites collected from SFM (Table 5 and Table 6). With the exception of agricultural lands, where low abundance did not allow for comparative analysis, all significant results occurred in the fall, when capture rates of SFM were significantly higher. The MI of chigger mites was higher in grasslands (96.43) than that in forests (75.00), while no significant difference in the ecotones (96.77) from forests and grasslands were observed. These results were consistent with those of the IR and MI of the dominant species, *L. pallidum*.

### 3.3. Chigger Mites Parasitizing Small Mammals by Habitat and Season

**Figure 5:**
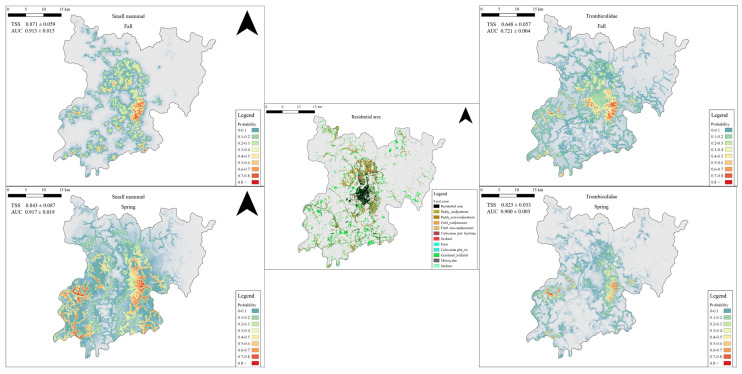
Prediction map of both small mammals and Trombiculidae by season (upper left: small mammals in fall, upper right: Trombiculidae in fall, center: residential area, bottom left: small mammals in spring, bottom right: Trombiculidae in spring).

The predicted distribution area of small mammals, based on MaxEnt analysis, was larger in the spring (144.68 km²), when fewer individuals were captured, and smaller in the fall (52.05 km²), when more individuals were captured. The overlap between the predicted distribution area of small mammals with an occurrence probability of 0.6 or higher and urban and agricultural lands was 16.66 km² (11.51%) in the spring and 5.84 km² (11.22%) in the fall, indicating a larger overlap in the spring. In contrast, the distribution of chigger mites parasitizing small mammals was broader in the fall (35.39 km²) than in the spring (15.36 km²), with the overlap in areas with an occurrence probability of over 0.6 also being greater in the fall (5.5 km²), showing different patterns compared to their small mammal hosts (Table 7; Figure 5).

## 4. Discussion

Small mammal communities in fragmented habitats are known to vary in species composition and abundance [58,59]. In this study, the species richness indices for the four habitat types were highest in forests, followed by ecotones, grasslands, and agricultural lands. Forests farther away from human settlements have the largest patches and coexist with a variety of landscape types, such as valleys, woodlands, and shrublands, resulting in the greatest number of species captured and a lower proportion of dominant species, which is likely to contribute to higher small mammal species richness than that by the other habitats. Despite their close proximity to human settlements, ecotones are characterized by a combination of different landscape elements that allow for a wide range of species to be supported by a variety of vegetation types [60], including herbaceous and shrubby vegetation. Contrarily, grasslands are characterized by a monotonous vegetation type consisting of only short-lived herbaceous species, with small patches such as bare patches and set-aside fields, which are likely to explain the low species richness due to the high dominance of one species, SFM.

The Striped field mouse is known to be the most dominant small mammal species in a variety of habitat types in the ROK [47,61] and accounted for 76.8% of the six small mammal species captured in this study, with the highest proportion in all the four habitat types. Of the small mammals in the three habitats excluding agricultural land, SFM accounted for 61%, 80%, and 84% in forests, ecotones, and grasslands, respectively. Given that the comparison of the distance between capture sites and human settlements showed that capture sites in forests were significantly farther away from human settlements and that SFM capture rates and dominance were significantly lower in forests, this suggests that SFM is capable of densely populating habitats in vegetation types adjacent to human settlements.

Captured in four habitat types during both spring and fall seasons, SFM is a known host for several diseases in addition to scrub typhus, including hantavirus and severe fever with thrombocytopenia syndrome (SFTS) [62,63,64,65,66,67]. All ectoparasites from the captured SFM belonged to four families and one order, including all eight species within the family Trombiculidae. Excluding the IRs of EHM and SC, which have fewer captured individuals, and the MI of EHM, which has a large standard error, they can be considered the main hosts of ectoparasites with the highest IRs and MIs.

Consistent with the earlier study reporting that the MI of chigger mites was higher in areas with simpler landscape elements resulting in lower species diversity of host communities and higher abundance of dominant species [68], this study found that the MI of chigger mites on SFM was significantly higher in grasslands (215.41 ± 20.70) and ecotones (171.67 ± 30.33), with high dominance of SFM, than that in forests (76.67 ± 32.11). This may have been due to the dominance and high density of SFM in the vicinity of urban lands, resulting in increased host encounter opportunities for chigger mites. Comparative analysis of the IR and MI across habitats showed significantly lower values in forests, where SFM was less dominant, and significantly higher values in grasslands and ecotones, where SFM was more dominant. Given that the MI of parasites increases with the abundance of SFM, the prevalence of the dominant chigger mite species, *L. pallidum*, appears to be influenced by the dominance and abundance of SFM among small mammals.

The predicted distribution of small mammals using the MaxEnt model showed a narrow, dense distribution in the fall and a wide, open distribution in the spring. The distribution in 6h4 fall was predicted to be narrow, centered on grasslands and ecotones, influenced by the large number of captured SFM, while the distribution in the spring was predicted to be wide, reflecting the characteristics of the four habitat types as the number and dominance of captured SFM in grasslands and ecotones decreased. The predicted distribution area of chigger mites was narrower in the spring and wider in the fall, in contrast to that of the results for small mammals. This may have been to be related to higher IR and MI in the fall, and given that an area of 0.54 km^2^ was present with a probability of occurrence greater than 0.6, it seemed to represent a weighting by the low abundance of chigger mites in the spring.

Research aimed at the prevention of scrub typhus (*Orientia tsutsugamushi*), which is widely distributed across the Asia–Pacific region, has explored various aspects of the relationships between small mammal hosts, chigger mite vectors, and factors such as seasonality and habitat [69,70,71,72,73,74]. Despite the limitations of a relatively small scale and short duration, this study is significant in examining the dominance of SFM, which is widely distributed across Eurasia [75,76], as a potential primary host for chigger mites. Additionally, by predicting seasonal chigger mite distribution based on host dominance and abundance, this study identifies key risk areas within human residential areas. These findings are expected to make a valuable contribution to future efforts in disease prevention and epidemiological research.

## 5. Conclusions

Conclusively, the abundance of chigger mites is influenced by the dominance of SFM as the primary host in the habitat. The dense distribution of the dominant species was found to be in grasslands and ecotones near human settlements dominated by SFM, and the densities were thought to be higher in the fall in relation to the life cycle of chigger mites and the seasonal densities of their hosts. Therefore, the habitat disturbance by human interference may increase the population and distribution area of SFM and expand its habitat. Hence, if diversification of the vegetation community within a habitat, such as by planting trees, increases the richness of the small mammal community and reduces the dominance of SFM, the abundance of chigger mites is likely to decrease. Additionally, measures such as set-aside field management and compartmentalization of agricultural lands and forests to reduce grasslands and ecotones, which are the preferred habitats of SFM, are expected to suppress the dispersal of chigger mites near human settlements.

## Figures and Tables

**Table 1:** Variables used for modeling the presence of small mammals and ectoparasites by season.

No.	Description	No.	Description
1	distance to residential area	12	distance to mixed forest
2	distance to paddy_readjustment	13	distance to grassland_natural
3	distance to paddy_non-readjustment	14	distance to grassland_etc
4	distance to field_readjustment	15	distance to wetland_inland
5	distance to field_non-readjustment	16	distance to riverside
6	distance to cultivation plot_facilities	17	distance to rock
7	distance to orchard	18	distance to mining site
8	distance to farm	19	distance to stadium
9	distance to cultivation plot_etc	20	distance to bareland_etc
10	distance to broadleaf forest	21	distance to stream
11	distance to coniferous forest	22	distance to lake

**Table 2:** Capture and infestation rate by season and habitats of all small mammal species.

Host Species		Habitats							
		Agricultural Land		Ecotone		Forest		Grassland	
	(%)	Fall	Spring	Fall	Spring	Fall	Spring	Fall	Spring
SFM	Capture rate	0.3	0.8	a* 5.2	b* 2.2	2.0	1.5	a* 9.3	b* 2.5
	Infestation rate		0.5	5.2	1.8	1.8	1.5	7.7	2.5
UWS	Capture rate	0.8		0.5	0.2	0.3		1.2	0.2
	Infestation rate	0.2		0.5		0.3		0.3	0.2
ALWS	Capture rate		0.2	0.3		0.7	0.2	0.3	
	Infestation rate		0.2	0.3		0.5		0.2	
KRV	Capture rate				0.2		0.3	0.3	0.2
	Infestation rate				0.2		0.3	0.2	
EHM	Capture rate			0.2		0.5			
	Infestation rate			0.2		0.5			
SC	Capture rate				0.5	0.2			
	Infestation rate				0.5	0.2			
H’		0.918		0.794		1.231		0.574	
RI		1.853		2.873		3.265		1.559	
E’		0.836		0.443		0.687		0.414	
Capture rate (%)		a** 2.0		b** 9.2		bc** 5.7		d** 14.0	
Infestation rate (%)		a** 33.3		b** 94.5		b** 91.2		b** 86.90	

* Different letters indicate significantly different between season (*p* < 0.05); ** different letters indicate significantly different among habitat type (*p* < 0.0083).

**Table 3:** Capture and infestation rate by season and habitats of SFM.

SFM	Season		Habitats			
	Fall	Spring	Agricultural Land	Ecotone	Forest	Grassland
Captured individual	101	41	6	44	21	71
Infested individual	96	38	3	42	20	69
Capture rate	^a^* 8.42	^b^ 3.42	^a^** 1.00	^b^ 7.33	^c^ 3.50	^d^ 11.82
Infestation rate	95.05	92.68	50.00	95.45	95.24	94.37
Dominance rate	75.93	78.84	^a^ 50.00	^bc^ 80.00	^a^ 61.76	^bc^ 84.52

* Different letters indicate significantly different between season (*p* < 0.05); ** different letters indicate significantly different among habitat type (*p* < 0.0083).

**Table 4:** Infestation rate (%) and mean intensity among infected host species (*n* = number of host / IR = infestation rate / MI = mean intensity).

		Trombiculidae		Ixodidae		Ctenophthalmidae		Laelapidae		Anoplura	
Host Species	*n*	IR(%)	MI	IR(%)	MI	IR(%)	MI	IR(%)	MI	IR(%)	MI
SFM	126	88.1	137.5 ± 13.6	39.7	2.6 ± 0.3	24.6	2.2 ± 0.3	57.1	5.8 ± 1.0	10.3	1.9 ± 0.4
UWS	6	83.3	16.2 ± 12.8	33.3	3.0 ± 2.0	16.7	2.0				
ALWS	9	33.3	3 ± 1.5	77.8	4.6 ± 2.2	44.4	1.3 ± 0.3				
KRV	3	100	250.3 ± 115.5			66.7	2.0			33.3	1.0
EHM	4	50.0	89.5 ± 84.5	50.0	1.0	25.0	6.0	50.0	2.5 ± 1.5		
SC	4	100	41.5 ± 28.9	25.0	68.0	25.0	1.0				
Total	152	84	127.0 ± 13.2	40.8	3.8 ± 11.0	26.3	2.2 ± 0.3	48.7	5.7 ± 1.0	9.2	1.8 ± 0.4

**Table 5:** Mean intensity of Trombiculidae parasitic on SFM in fall.

		Ecotone (*n* = 31)	Forest (*n* = 12)	Grassland (*n* = 56)
Trombiculidae	IR	96.77	75.00	96.43
	MI	171.67 ± 30.33	76.67 ± 32.11	215.41 ± 20.70
*L. orientale*	IR	67.74	75.00	62.50
	MI	11.81 ± 1.60	8.89 ± 5.84	10.86 ± 1.69
*L. palpale*	IR	45.16	50.00	64.29
	MI	15.71 ± 5.13	11.33 ± 7.76	10.22 ± 2.01
*L. pallidum*	IR	96.77	66.67	96.43
	MI	141.33 ± 29.32	26.25 ± 21.84	183.15 ± 16.61
*L. zetum*	IR	41.94	25.00	3.57
	MI	10.67 ± 3.03	20.67 ± 28.87	6.00 ± 2.00
*N. gardellai*	IR	19.35 ^a^	25.00 ^a^	3.57 ^b^
	MI	6.33 ± 3.95	4.67 ± 3.06	5.00 ± 1.00
*N. japonica*	IR	-	8.33	-
	MI	-	6.00	-
*N. nagayoi*	IR	48.39	50.00	48.21
	MI	8.00 ± 1.62	12.67 ± 10.01	24.00 ± 16.93
*N. tamiyai*	IR	29.03	8.33	21.43
	MI	6.22 ± 1.58	16.00	8.83 ± 3.89
*E. koreanensis*	IR	32.26	66.67	21.43
	MI	10.00 ± 3.49	19.75 ± 29.11	18.17 ± 7.41

Different letters indicate significantly different between season (*p* < 0.05).

**Table 6:** Mean intensity of Trombiculidae parasitic on SFM in spring.

		Ecotone (*n* = 13)	Forest (*n* = 9)	Grassland (*n* = 15)
Trombiculidae	IR	69.23	88.89	73.33
	MI	37.56 ± 22.07	49.00 ± 14.16	111.45 ± 33.95
*L. orientale*	IR	53.85	55.56	60.00
	MI	6.00 ± 1.90	20.00 ± 4.24	25.56 ± 13.22
*L. palpale*	IR	7.69	33.33	26.67
	MI	4.00	16.00 ± 6.00	24.50 ± 15.35
*L. pallidum*	IR	30.77	11.11	46.67
	MI	16.50 ± 9.39	2.00	108.00 ± 41.59
*L. zetum*	IR	23.08	77.78	46.67
	MI	75.33 ± 57.18	34.29 ± 14.69	18.86 ± 7.48
*N. gardellai*	IR	-	-	-
	MI	-	-	-
*N. japonica*	IR	-	-	-
	MI	-	-	-
*N. nagayoi*	IR	-	-	20.00
	MI	-	-	2.00 ± 0.00
*N. tamiyai*	IR	-	-	-
	MI	-	-	-
*E. koreaensis*	IR	-	11.11	13.33
	MI	-	2.00	2.00 ± 0.00

**Table 7:** The area (km^2^) of the presence probability of Trombiculidae and small mammals.

	Season	Predicted Area		Ratio of Overall Residential Area (%)
		Presence	Overlapped with Residential Area (Probability > 0.6)	
Small mammals	Fall	52.05	5.84	14.31
	Spring	144.68	16.66	40.84
Trombiculidae	Fall	35.39	5.5	13.48
	Spring	15.36	0.54	1.32

## Data Availability

The data presented in this study are available on request from the corresponding author.
